# Using the modified Delphi method to establish clinical consensus for the diagnosis and treatment of patients with rotator cuff pathology

**DOI:** 10.1186/s12874-016-0165-8

**Published:** 2016-05-20

**Authors:** Breda H. Eubank, Nicholas G. Mohtadi, Mark R. Lafave, J. Preston Wiley, Aaron J. Bois, Richard S. Boorman, David M. Sheps

**Affiliations:** Department of Health and Physical Education, Faculty of Health, Community, and Education, Mount Royal University, 4825 Mount Royal Gate SW, Calgary, AB T3E 6K6 Canada; Sport Medicine Centre, Faculty of Kinesiology, University of Calgary, 2500 University Drive NW, Calgary, AB T2N 1N4 Canada; Orthopaedic Surgeon, Sport Medicine Centre, University of Calgary, 2500 University Drive NW, Calgary, AB T2N 1N4 Canada; Division of Orthopaedics, Department of Surgery, University of Alberta, 116 St & 85 Ave., Edmonton, AB T6G 2R3 Canada

**Keywords:** consensus guideline, Delphi technique, experts, rotator cuff, algorithm, clinical practice guideline

## Abstract

**Background:**

Patients presenting to the healthcare system with rotator cuff pathology do not always receive high quality care. High quality care occurs when a patient receives care that is accessible, appropriate, acceptable, effective, efficient, and safe. The aim of this study was twofold: 1) to develop a clinical pathway algorithm that sets forth a stepwise process for making decisions about the diagnosis and treatment of rotator cuff pathology presenting to primary, secondary, and tertiary healthcare settings; and 2) to establish clinical practice guidelines for the diagnosis and treatment of rotator cuff pathology to inform decision-making processes within the algorithm.

**Methods:**

A three-step modified Delphi method was used to establish consensus. Fourteen experts representing athletic therapy, physiotherapy, sport medicine, and orthopaedic surgery were invited to participate as the expert panel. In round 1, 123 best practice statements were distributed to the panel. Panel members were asked to mark “agree” or “disagree” beside each statement, and provide comments. The same voting method was again used for round 2. Round 3 consisted of a final face-to-face meeting.

**Results:**

In round 1, statements were grouped and reduced to 44 statements that met consensus. In round 2, five statements reached consensus. In round 3, ten statements reached consensus. Consensus was reached for 59 statements representing five domains: screening, diagnosis, physical examination, investigations, and treatment. The final face-to-face meeting was also used to develop clinical pathway algorithms (i.e., clinical care pathways) for three types of rotator cuff pathology: acute, chronic, and acute-on-chronic.

**Conclusion:**

This consensus guideline will help to standardize care, provide guidance on the diagnosis and treatment of rotator cuff pathology, and assist in clinical decision-making for all healthcare professionals.

**Electronic supplementary material:**

The online version of this article (doi:10.1186/s12874-016-0165-8) contains supplementary material, which is available to authorized users.

## Background

Rotator cuff pathology can be defined as acute or chronic tears of the rotator cuff, and are the most common cause of shoulder pain seen by physicians [1–4]. Rotator cuff pathology also ranks second in work-related injury, and is the second most common reason for lost time from physically demanding occupations [3, 5–7]. The incidence rate of rotator cuff pathology increases with age and is frequently a bilateral disease [8]. Additionally, although rotator cuff tears can initially be asymptomatic, the literature has shown that shoulder pain often develops in individuals within 5 years of the injury [3]. For patients with symptoms, rotator cuff pathology can be long-lasting, debilitating, and costly (i.e., both direct and indirect costs) [5, 6]. Therefore, it is essential that patients have access to timely and high quality care.

High quality care (i.e., the ideal state of care) occurs when a patient comes into contact with their respective healthcare system and the system is seen as accessible, appropriate, acceptable, effective, efficient, and safe [9]. Anecdotal evidence has suggested that patients presenting to the healthcare system with rotator cuff pathology experience less than ideal quality care plagued by lengthy wait times, challenges in coordinating care, and inefficient use of healthcare resources. Evidence-informed literature has suggested that improper management of musculoskeletal disorders can result in chronic conditions that last several years and result in significant costs to both the patient and healthcare system [10, 11]. Subsequently, management of rotator cuff pathology is in need of quality improvement through evidence-informed decision-making.

Evidence has shown that the use of consensus guidelines improves the quality of healthcare provided by recommending evidence-based best practice care [12, 13]. A systematic review, conducted by the authors, initially identified four existing guidelines related to the management of rotator cuff pathology. Beaudreuil et al. [14] developed a consensus guideline for the management of rotator cuff tears but focused on indications for surgery and surgical techniques. Hopman et al. [15] created a consensus guideline for the management of rotator cuff syndrome, but only focused on recommendations to improve clinical outcomes for injured Australian workers aged 18 – 65. Furthermore, several of their recommendations were limited to the workplace environment only. The American Academy of Orthopaedic Surgeons (2010) published a guideline for optimizing the management of rotator cuff problems [16]. However, of the 31 recommendations made by the working group, 19 were determined to be ‘inconclusive’ because of the absence of definitive evidence. Additionally, of the recommendations that reached consensus, only four statements were classified as moderate-grade evidence, while the remaining were classified as weak or lower [16]. Oliva et al. [17] published a systematic review of clinical practice discussing “18 hot topics involved in rotator cuff tears”. Although evidence from the literature search was presented on potential management strategies from etiopathogenesis to surgical treatment, no direct recommendations or decision tree approaches to treatment were made. Wait time benchmarks for clinician consultation, specialist treatment, and diagnostic imaging for patients presenting with rotator cuff pathology were also not presented in any of the guidelines. Subsequently, there are currently no comprehensive Canadian guidelines for clinicians who treat patients with rotator cuff pathology or clinical pathway algorithms, which include benchmark timelines that could outline patient care within appropriate timeframes. Therefore, the purpose of this study was twofold: 1) to develop a clinical pathway algorithm that sets forth a stepwise process for making decisions about the diagnosis and treatment of rotator cuff pathology presenting to primary, secondary, and tertiary healthcare settings; and 2) to establish clinical practice guidelines for the diagnosis and treatment of rotator cuff pathology to inform decision-making processes within the algorithm. Consensus was developed around five clinical domains: screening, diagnosis, physical examination, investigations, and treatment. This guideline was intended to support clinical decision-making by healthcare professionals for patients presenting with rotator cuff pathology.

## Methods

The consensus process incorporated a three-step modified Delphi method [18, 19], which took place between January and April 2015. The Delphi method is recommended for use in the healthcare setting as a reliable means of determining consensus for a defined clinical problem [20–25]. This method is an iterative process that uses a systematic progression of repeated rounds of voting and is an effective process for determining expert group consensus where there is little or no definitive evidence and where opinion is important [21]. Initially, a comprehensive list of items was identified and consensus was built from the feedback provided by expert participants from the preceding rounds. The modified Delphi method consisted of two rounds of email questionnaires and a final face-to-face meeting. The final face-to-face meeting was not a component of the original Delphi method developed by Dalkey and Helmer [19] in 1963; rather, it was adopted from the modified Ebel procedure [26–28] and is also known as the Estimate-Talk-Estimate process [29]. The modified Delphi method was chosen because it allowed for expert interaction in the final round. This allowed members of the panel to provide further clarification on some matters and present arguments in order to justify their viewpoints. Studies have demonstrated that the modified Delphi method can be superior to the original Delphi method and perceived as highly cooperative and effective [29, 30]. This technique is also often used in the health field for helping groups of experts develop multi-attribute models [31]. Since a goal of this study was to develop a clinical pathway algorithm, the final face-to-face meeting facilitated the development of decision tree approaches to healthcare treatment that would have been difficult to complete using original Delphi methodology. Ethics approval for this study was provided by the Conjoint Health Research Ethics Board at the University of Calgary.

### Panel selection

Although five to ten experts are considered adequate for content validation [32], fourteen experts were initially contacted and asked to participate in consensus development. All 14 provided consent and agreed to participate. Clinical decisions on individual patients should be based on scientific evidence and the clinical experience of the healthcare provider [17]. Therefore, experts were chosen to represent professional groups that directly influence patient care and would benefit from clinical practice guidelines. Since the goal of the study was to develop standards of care based on scientific information and medical advice, patients were not included as panel experts. Panel members were identified from the two largest cities in Alberta, Canada and selected based on their clinical and research expertise in the evaluation and treatment of patients with rotator cuff pathology. The panel consisted of athletic therapists, physiotherapists, sport medicine physicians, and orthopaedic surgeons. Once panel members were contacted, the goals and processes of the project were explained and consent was obtained.

### Systematic review of the literature

A systematic review of the literature was performed to identify best practice evidence for clinical guideline development. MEDLINE from 1946 to January 2015 was searched for English-language literature. The search strategy combined headings and keywords for “rotator cuff” and “screening” or “diagnosis” or “treatment” or “physical examination” or “management”. Grey literature was also searched.

A sub-group of three experts from the panel formed a core group. A member of the core group screened all titles and abstracts to discard irrelevant ones. Articles from the literature search were included if they defined, described, or recommended appropriate clinical information related to rotator cuff pathology including: 1) screening questions to rule out underlying pathologies that required different care pathways; 2) history-taking questions that aided in the differential diagnosis of shoulder pain; 3) physical examination and special tests; 4) indications for diagnostic investigations; and 5) treatment. Articles discussing specific surgical techniques were excluded. Reference lists from included publications were also screened to identify additional papers. All members of the core group independently completed a second screening to verify the completeness of the initial list. Additional references were provided by the core group. Full texts of relevant studies were retrieved and reviewed for eligibility.

### Data extraction and statement development

Full-text publications were searched for best practice evidence to be used in clinical guideline development. Relevant information was collected from included studies regarding best practices on how to screen, diagnose, treat, and perform physical examinations on patients presenting with rotator cuff pathology. Publications were also searched for best practice diagnostic guidelines for imaging. Information was extracted and used to develop consensus statements. Statements were compiled into a Microsoft Excel (2007) spreadsheet. Each statement was assigned the highest level of evidence available based on the systematic review of the literature to categorize the quality of each statement, and to aid in clinical decision-making. Levels of evidence were adapted from Wright et al. [33] and ranged from randomized controlled trials (Level 1) to expert opinion (Level 5) (Table 1). Members of the core group reviewed the Excel spreadsheet, and subsequently met as a group to discuss discrepancies and finalize a draft consensus document.

**Table 1:** Levels of evidence

1	Evidence obtained from: • systematic review of randomized controlled trials; • high-quality randomized controlled trials; • high-quality prospective studies (e.g., all patients were enrolled at the same point of their disease with 80 % follow-up of enrolled patients); or • testing of previously developed diagnostic criteria in series of consecutive patients.
2	Evidence obtained from: • systematic review of level 2 studies or level 1 studies with inconsistent results; • lesser quality randomized controlled trials (e.g., < 80 % follow-up, no blinding, or improper randomization); • prospective comparative studies; • retrospective studies; • lesser quality prospective studies (e.g., patients enrolled at different points in their disease or <80 % follow-up); or • development of diagnostic criteria on the basis of consecutive patients (with universally applied reference gold standard).
3	Evidence obtained from: • systematic review of level 3 studies; • case control studies; • retrospective comparative studies; or • study of nonconsecutive patients (without consistently applied reference gold standard).
4	Evidence obtained from: • case series; or • case control study with poor reference standard.
5	Evidence obtained from: • expert opinion.

### Round 1

The draft document containing the list of statements was circulated by email to all 14 panel members accompanied by a clear explanation of the objectives of the study and specific instructions for member participation. Each expert was asked to vote by marking “agree” or “disagree” beside each statement. Experts were also given the opportunity to provide comments and suggest additional items that may not have been included when developing the initial list of statements. In round 1, the intention was also to clarify any redundancy or issues regarding comprehension or syntax of each statement. Response frequencies for each item were calculated and entered anonymously into a database by a research assistant. Statements required 80 % agreement from the panel (i.e., agreement among 11 of 14 experts) in order to accept or omit a statement during construction of the final guideline. In other words, if 11 experts agreed on a statement, the statement was accepted for the final guideline document; if 11 disagreed, the item was omitted from the list of statements. Eighty percent was chosen as an appropriate cut off based on work by Lynn [32], who suggested that at least 80 % of experts must agree on an item in order to achieve content validity when there are at least 10 experts participating in consensus development. Statements not meeting 80 % agreement were modified according to feedback provided by the expert panel and redistributed to the panelists for round 2.

### Round 2

The list of statements that did not meet consensus from round 1 was emailed to all 14 members. In round 2, the experts used the same voting method as described for round 1, but with the knowledge of the group scores and comments. Thus, participants could reflect upon the group results and change their mind, while preserving the anonymity of their responses. Final responses were analyzed as described for round 1, and statements not meeting expert agreement were retained for discussion in round 3.

### Round 3

Round 3 comprised of a face-to-face meeting. Eighty percent agreement was still used to determine acceptance or rejection of a statement. Round 3 voting occurred using a show of hands and anonymity was not retained. Panel members were encouraged to discuss the remaining statements until agreement was reached to retain, modify, or eliminate the statement from the final guideline document. Once full consensus was reached on statements for the final guideline document, the panel spent the remainder of the face-to-face meeting to determine the optimal clinical pathway for patients presenting with rotator cuff problems. The goal was to develop decision tree approaches to healthcare treatment for patients presenting with rotator cuff pathology. Members of the panel identified three types of rotator cuff pathology currently presenting to the healthcare system: acute, chronic, and acute-on-chronic rotator cuff tears. The face-to-face meeting was mediated by a facilitator, which focused discussions around the flow of patients through primary, secondary, and tertiary healthcare settings. Specifically, panel members were asked to produce a map of the patient’s journey through the healthcare system by outlining the sequence of steps and activities performed at each step. Panel members were also told to establish benchmark timelines for initial consultations by healthcare practitioners, specialist care, and diagnostic imaging. The goal was to develop an algorithm in which clinicians could follow in order to get patients to the right people, in the right order, in the right place, within the right timeframe, and with the right outcomes.

## Results

### Panel selection

Fourteen experts representing the two largest cities in Alberta, Canada (i.e.*,* Edmonton and Calgary), formed the expert panel. All 14 experts participated in rounds 1 and 2. Only 11 experts could attend the face-to-face meeting; of which one expert attended the meeting via video conference and was only able to participate for half of the meeting. The remaining three experts were unable to attend the meeting due to conflicting obligations.

### Systematic review of the literature

The literature search was performed to identify best practice evidence for clinical practice guidelines. The search identified 1300 publications. Of these, 112 were selected based on titles and abstracts, an additional 23 publications were identified from reference lists, and two were provided by the core group. In all, 137 publications relating to screening, diagnosis, physical examination, investigations, and treatment of rotator cuff pathology were included.

### Data extraction and statement development

For the development of screening and diagnostic statements, information was compiled from studies that proposed history-taking questions that could be used to identify or differentially diagnose rotator cuff pathology. For the development of statements pertaining to physical examination of patients presenting with rotator cuff symptoms, information was compiled from studies that discussed the use of observation, range of motion, special tests, and palpation in examination procedures. For the development of statements pertaining to investigations, information was retrieved pertaining to best practice diagnostic imaging guidelines for patients presenting with rotator cuff pathology symptoms. For statements pertaining to treatment of rotator cuff pathology, the following information was compiled: indications for non-operative management; indications for surgical management; benchmarks to treatment; and best practices with respect to exercise programs. Evidence from included publications generated 123 statements (i.e.*,* clinical practice guidelines) for patients presenting with rotator cuff pathology. Evidence from pre-existing guidelines identified by the literature search was also cross-referenced during the development of consensus statements to ensure that the highest level of evidence was achieved. The 123 statements were circulated to all members of the expert panel for round 1 voting.

### Round 1

**Fig. 1:**
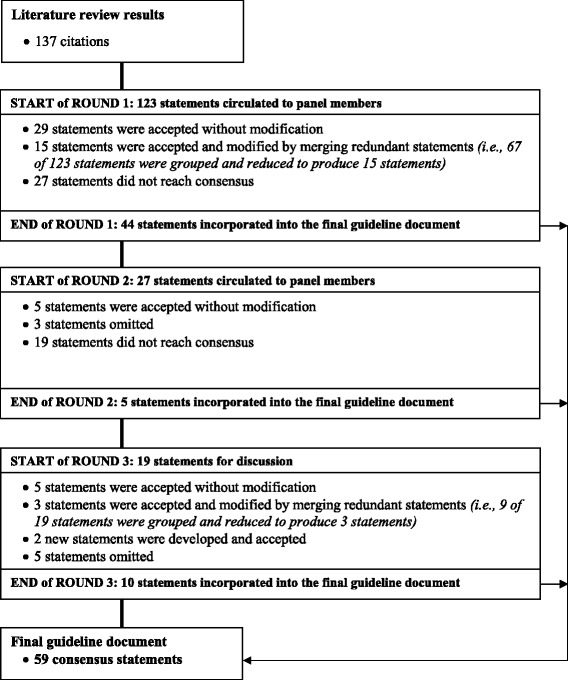
Modified Delphi methodology and results

After round 1 voting was completed and comments were summarized, redundant statements and statements sharing similar constructs were grouped and reduced. Specifically, 67 of 123 initial statements were combined and reduced to produce 15 statements that reached consensus, and were accepted for the final document. For example, the following five items were originally included in the list of statements for round 1 (physical examination domain): 1) active range of motion for the shoulder should be performed bilaterally; 2) active range of motion for the shoulder should be assessed for external rotation at 0° of abduction; 3) active range of motion for the shoulder should be assessed for shoulder internal rotation according to spinal level (i.e.*,* the highest vertebral level reached with the thumb extended); 4) assess shoulder elevation in the scapular plane; and 5) assess shoulder elevation in the sagittal plane. All five items received consensus (≥11 panel members voted “agree” on a statement), were combined into a single statement to reduce redundancy, and accepted for the final guideline document. The revised statement now reads “active range of motion for the shoulder should be performed bilaterally including: shoulder elevation in the scapular plane; shoulder elevation in the sagittal plane; external rotation at 0° abduction; and internal rotation according to spinal level (i.e.*,* the highest vertebral level reached with the thumb extended).” Twenty-nine of 123 initial statements were not deemed redundant, reached consensus (≥11 panel members voted “agree” on a statement), and were accepted into the final document without modification. In total, 44 statements from round 1 were accepted into the final guideline document. Twenty-seven of 123 initial statements did not reach consensus after round 1. Figure 1 illustrates the results of the modified Delphi method.

### Round 2

In round 2, 27 statements that did not receive consensus, along with accompanying comments that were made during round 1, were re-circulated to the panelists. After round 2 voting, panel members reached consensus on five of 27 statements that initially did not receive consensus in round 1 (≥11 panel members voted “agree” on a statement). These five statements were accepted into the final guideline document. The panel also reached consensus to omit three items from the final document (≥11 panel members voted “disagree” on a statement). Nineteen of 27 statements did not reach consensus after round 2.

### Round 3

The remaining 19 statements were reserved for discussion during the face-to-face meeting in round 3. Round 3 was used to seek clarification for statements that did not reach consensus in preceding rounds. It was also used to generate additional statements in cases where alternative practices reflected best practice. In round 3, five of 19 statements reached consensus after discussion (≥11 panel members voted “agree”), and were accepted into the final guideline document. Nine of nineteen statements were reduced into three statements because of redundancy. Two new statements were developed. The panel felt these two statements would serve to help clinicians in the differential diagnosis of rotator cuff pathology. In total, ten statements from round 3 were accepted into the final guideline document. Five of nineteen statements did not reach consensus after round 3 and were not included in the final guideline document. The final guideline document consists of 59 statements: 13 related to screening, 17 related to diagnosis, 18 related to physical examination, 4 related to investigations, and 7 related to treatment (Table 2).

**Table 2:** Clinical practice guideline for the diagnosis and treatment of rotator cuff pathology

Clinical domain	Consensus statement	Level of evidence^a^
Screening	The following thirteen questions should be included during history taking to determine a patient’s relative priority of need and the proper place of treatment:	
	• How old are you [73]?	2
	• Do you smoke [74]?	3
	• Did your problem occur at work or because of a work-related incident (i. e., Is this Workers’ Compensation Board related) [75]?	2
	• Is your shoulder problem part of an active medicolegal or third party claim [76]?	2
	• Do you have neck pain? If yes, is this separate from your shoulder pain [77]?	2
	• Do you have unexplained sensory deficits in your arm, wrist, or hand (i.e., numbness, tingling, burning) [77]?	2
	• Do you have other upper limb pain? If yes, are you experiencing pain in the forearm, elbow, wrist, or hand [78–80]?	2-3
	• Is your shoulder problem associated with fevers, chills, and/or weight loss [77]?	2
	• Are you currently receiving treatment at a chronic pain clinic? If yes, is your shoulder problem part of a generalized pain condition [81]?	2
	• Are you currently receiving active treatment for a generalized joint condition (e.g., arthritis involving multiple joints in your body)? If yes, is this affecting your current shoulder problem [82]?	3
	• Are you currently receiving active treatment for a neurological/neuromuscular condition (e.g., stroke, multiple sclerosis)? If yes, is this affecting your current shoulder problem [77]?	2
	• Are you currently receiving active treatment for a diagnosis of cancer? If yes, is this affecting your current shoulder problem [77]?	2
	• Are you currently receiving active treatment for a medical condition such as diabetes, renal disease, respiratory disease, or ischemic heart disease? If yes, is this affecting your current shoulder problem [77]?	2
Diagnosis	The following seventeen questions should be included during history-taking to confirm rotator cuff pathology and/or rule out other conditions:	
	• What is your sex [83]?	2
	• What is your dominant hand [77]?	2
	• What is your occupation [77]?	2
	• When did you first notice you had shoulder pain or a problem with your shoulder [77]?	2
	• Do you have pain in your shoulder [77]?	2
	• Is your shoulder pain a result of a specific injury? If yes, describe how you injured your shoulder in as much detail as possible [84]?	2
	• Can you characterize your pain including: date; severity; onset during activity, onset during overhead activity; presence of night pain; presence of pain at rest [77, 79]?	2-3
	• Does anything help to relieve the pain? If yes, please specify [79]?	2
	• Where do you feel the most pain (i.e., top, side, front, back of shoulder) [85]?	3
	• Does your shoulder feel stiff [79]?	
	• Does your shoulder feel loose or unstable [77]?	2
	• Does your shoulder come out of place [77]?	2
	• Does your shoulder dislocate [77]?	2
	• Has your shoulder dislocated in the past [77]?	2
	• Do you hear or feel unusual sensations such as catching, locking, or grinding in your shoulder joint [86]?	2
	• Do you have painful clicking, grinding, or clunking in your shoulder [86]?	2
	• Does your shoulder feel weak [86]?	2
Physical Examination	The following eighteen items should be included during a physical examination to confirm rotator cuff pathology and/or rule out other conditions:	
	• In observing the patient, the shoulder should be exposed and observed from the front and back [85].	3
	• Active range of motion for the cervical spine should be performed for all planes (i.e., flexion, extension, side flexion, rotation) [87]	2
	• Active range of motion for the shoulder should be performed bilaterally including: shoulder elevation in the scapular plane; shoulder elevation in the sagittal plane; external rotation at 0 degrees abduction; and internal rotation at the spinal level (i.e., the highest vertebral level reached with the thumb extended) [85, 87, 88].	2
	• Range of motion should be assessed for a painful arc [89].	2
	• Scapulohumeral rhythm should be assessed for scapular dyskinesis [90].	2
	• Passive range of motion should only be assessed if active range of motion is limited [85].	3
	• If active range of motion is limited, assess shoulder using external rotation lag sign and Hornblower’s sign [91, 92].	2
	• If active and passive ranges of motion are limited, assess isolated glenohumeral joint range of motion [93].	2
	• If adhesive capsulitis is suspected, bilaterally assess forward elevation and external rotation at 0 degrees abduction at the glenohumeral joint [94].	3
	• Palpation of the shoulder should occur at the point of maximum tenderness [89].	2
	• Manual muscle testing should be performed for the supraspinatus muscle in the scapular plane (i.e., thumb pointing down), and having the patient resist against a downward pressure placed on the forearms [95].	2
	• Manual muscle testing should be performed for the infraspinatus muscle by having the patient externally rotate from 45 degrees of internal rotation against resistance [96].	2
	• The Belly Press test should be used to assess subscapularis strength [97].	2
	• The Lift-off test should be used to assess subscapularis strength [98].	2
	• Neer’s impingement sign should be used to confirm impingement [99].	2
	• Hawkins-Kennedy sign should be used to confirm impingement [95].	2
	• Speed’s test should be used to confirm biceps muscle or tendon pathology [100].	2
	• Cross body adduction test should be used to rule out acromioclavicular joint sprain [87].	2
Investigations	The following four guidelines for investigations are recommended for patients that present with rotator cuff pathology:	
	• From a diagnostic and treatment perspective, a x-ray is a necessary test [49].	2
	• If rotator cuff disorder is suspected, the following x-ray views should be ordered at the initial visit: true anteroposterior view (Grashey view), axillary, and trans-scapular lateral [49, 101].	2
	• Ultrasound is the cost-effective investigation for defining a full-thickness rotator cuff tear [50].	2
	With respect to full-thickness rotator cuff tears, magnetic resonance imaging (MRI) is only required for surgical planning [102].	2
Treatment	The following seven guidelines for investigations are recommended for patients that present with rotator cuff pathology. These guidelines were expanded and merged to create clinical care pathways for three classifications of rotator cuff injuries: acute, chronic, and acute-on-chronic injuries.	
	*Acute rotator cuff pathology*	
	• Patients without pre-existing history of rotator cuff problems, presenting with an acute, traumatic injury (*i.e.,* definable traumatic event) of the rotator cuff resulting in dramatic loss of shoulder function, should be referred to a surgeon, and seen by the surgeon within 6 weeks after consultation with a primary care practitioner [103].	2
	*Chronic and acute-on-chronic rotator cuff pathology*	
	• All patients with chronic rotator cuff disorders should attempt a non-operative rotator cuff home exercise and stretch program [56, 104].	2
	• Patients presenting to healthcare professionals with chronic rotator cuff disorder should be prescribed a non-operative rotator cuff rehabilitation program at the initial visit, if one has not already been prescribed [56].	2
	• Stage 1 home programs (Weeks 0–6) should focus on decreasing shoulder pain and increasing shoulder range of motion through exercise, stretching, and high repetition movement patterns, four times every day (*i.e.*, pulley exercises, assisted range of motion for abduction, elevation, external rotation, internal rotation) [56].	2
	• Stage 2 home programs (Weeks 6–12) should focus on improving strength and muscular control at least once a day (*i.e*., banding exercises, scapular stabilizing exercises) [56].	2
	• Patients that are not able to achieve pain-free status with improved range of motion after 6 weeks should attempt additional pain control (*i.e*., cortisone injection) in adjunct to the non-operative rotator cuff home program [56].	2
	• Patients that fail a non-operative rotator cuff home program after 12 weeks should receive an ultrasound (*i.e*., the patient did not improve, remained symptomatic, elected to have surgery, and has not already had ultrasonography) [50, 56].	2

^a^Consensus statements were assigned the highest level of evidence available based on the systematic review. Evidence sources are listed in bracketed numbers after each statement. Levels of evidence were adapted from Wright et al. [33].

**Fig. 2:**
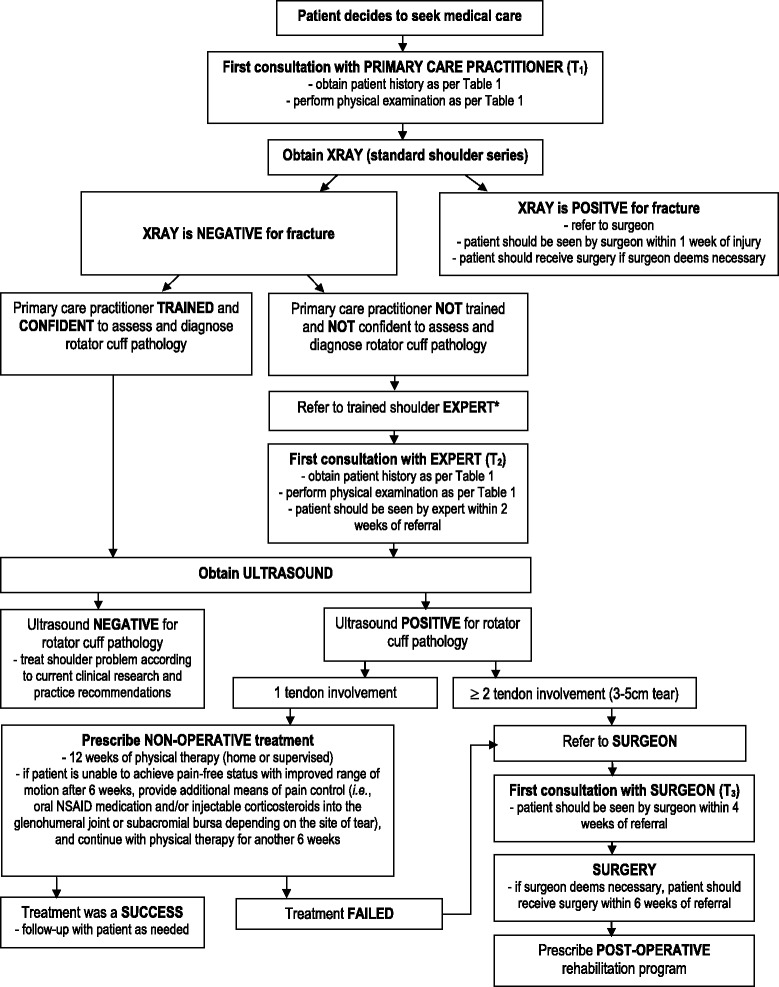
Clinical management algorithm for acute rotator cuff pathology. Legend: *A trained shoulder expert is any primary, secondary, or allied healthcare practitioner who has the expertise and knowledge in musculoskeletal medicine to appropriately and accurately assess shoulder pathology

**Fig. 3:**
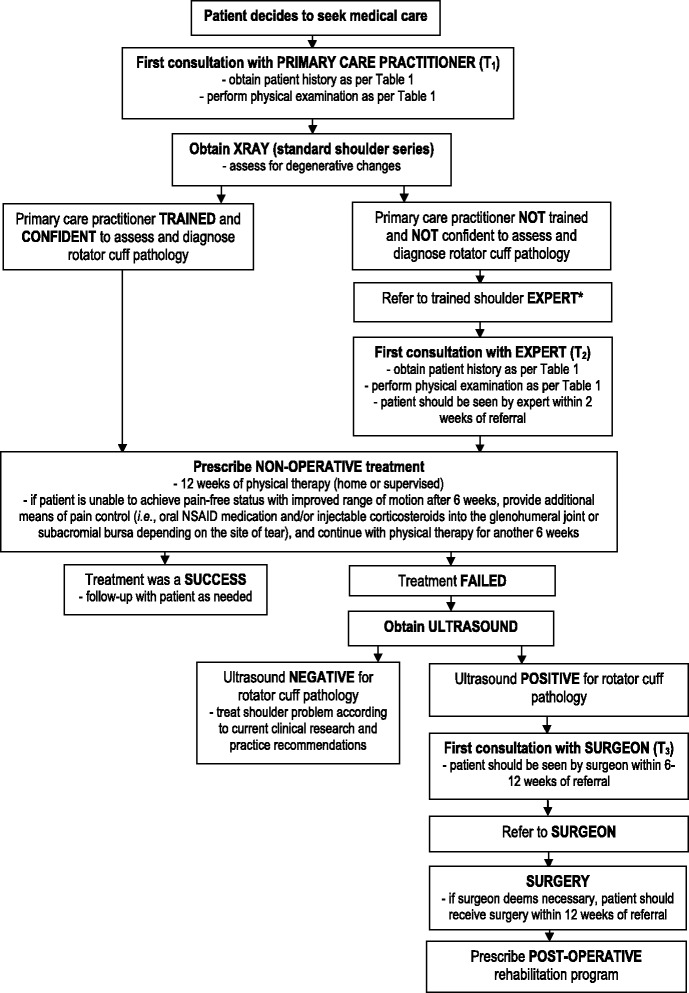
Clinical management algorithm for chronic and acute-on-chronic rotator cuff pathology. Legend: *A trained shoulder expert is any primary, secondary, or allied healthcare practitioner who has the expertise and knowledge in musculoskeletal medicine to appropriately and accurately assess shoulder pathology

The final face-to-face meeting was also used to establish clinical pathway algorithms. First, participating members identified and defined three distinct types of rotator cuff pathology: acute, chronic, and acute-on-chronic rotator cuff tears. Secondly, clinical pathway algorithms were developed for each type of rotator cuff pathology, incorporating decision tree approaches to treatment beginning with time of injury and ending with best practice guidelines for treatment. An acute rotator cuff tear was defined by the expert panel as a patient who presents with a discrete traumatic episode resulting in an injury to a previously asymptomatic shoulder. The panel recommended that patients be managed using the acute clinical care pathway if they meet the following inclusion criteria: active and high-functioning; asymptomatic prior to the event (i.e., no previous history of shoulder problems); a discrete traumatic tear attributable to a specific event or mechanism of injury; experiencing loss of function including an inability to lift the arm; and less than 65 years of age (Fig. 2). A chronic rotator cuff tear was defined as a patient that presents with shoulder pain of insidious or gradual onset or resulted from a previous traumatic episode. The panel recommended that these patients should be managed using the chronic clinical care pathway if they present with pain, weakness, and/or altered function (Fig. 3). An acute-on-chronic rotator cuff problem was defined as a patient with pre-existing rotator cuff pathology who experiences a traumatic episode to the ipsilateral shoulder. It was agreed that these patients should also be managed using the chronic clinical care pathway if they presented with pain, weakness, and/or altered function (Fig. 3). Results from the final meeting were summarized and distributed to the entire group for final remarks. The final document, including all steps in each clinical pathway algorithm, were accepted and reached unanimous agreement by the group. A description of each clinical pathway algorithm can be found in Additional file 1.

## Discussion

Rotator cuff pathology ranks among the most prevalent of musculoskeletal disorders, while treatment and management of this pathology is complex. In some cases, knowledge gaps exist as it pertains to clinical pathway algorithms and wait times benchmarks. In other cases, a multitude of treatment options exist for patients [34]. Thus, there is a need for evidence-based consensus where there is agreement. The current clinical care for patients in Canada is plagued with lengthy wait times, variations in quality and access to care, inefficient use of healthcare resources, lack of coordination between different disciplines and professional specializations, and physicians that are inadequately trained to manage musculoskeletal problems [35–40]. Development of algorithms and decision tree approaches to treatment provide a stepwise sequence that improves quality, consistency, and coordination of care across the entire continuum of care. This reduces costs by minimizing wait times; reduces inappropriate use of healthcare resources; and optimizes patient outcomes [41, 42]. Although four consensus guidelines were identified in a literature search, only Hopman et al. [15] contained algorithms with respect to managing shoulder pain in the Australian workplace. Therefore, this study implored the use of a modified Delphi method to develop clinical management algorithms for patients presenting to primary, secondary, and tertiary healthcare settings. The modified Delphi method was also used to build consensus around the diagnosis and treatment of patients with rotator cuff pathology. A detailed description of the Delphi method was included in this study to improve the quality of the final consensus guideline and to add a level of credibility to statement development and selection [43]. To our knowledge, this is the first use and reporting of a modified Delphi method to develop clinical pathway algorithms for patients presenting with rotator cuff pathology to healthcare settings.

Consensus was also reached for 59 statements representing five domains (e.g., screening, diagnosis, physical examination, investigations, and treatment) that could be used as clinical practice guidelines to inform decision-making processes within the algorithm. Screening and diagnostic questions are part of the history-taking portion of clinical care. Screening questions help to identify individual patients that require particular needs or alternative care pathways. Diagnostic questions help identify correct pathologies so that appropriate treatment decisions can be made. Utilization of appropriate screening and diagnostic questions can help improve the quality of patient care by accurately diagnosing problems and identifying correct pathologies. History-taking alone has been found to accurately diagnose clinical conditions 56 % to 82.5 % of the time [44–46].

Conducting an appropriate physical examination can also play a crucial role in the diagnosis of rotator cuff pathology. When the appropriate measures are performed, a physical examination is complementary to the history and should be used to confirm a diagnosis. In some instances, the physical examination can reveal unexpected diagnoses or add to information already collected [47]. Specifically, Hampton et al. [44] and Peterson et al. [45] were able to demonstrate that a physical examination can improve diagnostic accuracy by an additional 8.75 % (from 82.5 %) and 12 % (from 76 %) respectively.

Investigations can also play a role in diagnosing rotator cuff pathology. Advances in investigations, such as medical imaging, have provided healthcare professionals with new non-invasive tools to improve patient care [48]. Though physicians may possess different attitudes toward investigations, some rely on them more heavily than others [44]. Physicians should use the information collected from the history and physical examination to make diagnostic conclusions before relying on investigations. There are two justifications for any investigation. First, it should be used to answer a specific clinical question relating to diagnosis and management, but only when there is doubt regarding either; second, it can be used to measure the effect of treatment that cannot be assessed on symptoms or signs alone [46]. The modified Delphi method was used to reach consensus for three investigations: x-ray, ultrasound, and magnetic resonance imaging (MRI). In agreement with the literature, this guideline recommends that standard shoulder x-rays are necessary and cost-effective in any patient suffering from shoulder trauma, pain, or joint instability [49]. If additional investigations are warranted, an ultrasound examination is the cost-effective investigation for defining full-thickness rotator cuff tears. In 2009, de Jesus et al. [50] found ultrasound to be accurate in the diagnosis of both full and partial-thickness rotator cuff tears, and that ultrasound and MRI were comparable in both sensitivity and specificity. Although MRI is commonly ordered for this patient population, MRI is significantly more expensive, and in most cases, should be ordered by a surgeon primarily for surgical planning purposes. In a system where patients are waiting an average of 25 weeks in Alberta for MRI, patient care including treatment become delayed unnecessarily in cases where MRIs are not needed [51]. This recommendation is consistent with other musculoskeletal conditions such as lower back pain and acute knee injury [52, 53].

Clinical pathway algorithms ensure that patients receive the right care in the right place by the right person within the right timeframe [54]. The goal is to ensure patients are managed appropriately, efficiently, effectively, safely, and within acceptable timelines without wasting healthcare resources and worsening the health outcomes of patients. Clinical pathway algorithms were developed by the expert panel for three types of rotator cuff pathology: acute, chronic, and, acute-on-chronic (Figs. 2 and 3). Both algorithms detail essential steps in the care of patients throughout primary, secondary, and tertiary healthcare settings. Pathways are intended only as a guideline for practitioners and should be adapted to fit unique circumstances or based on the practitioner’s professional judgment. This guideline recommends that patients presenting to all healthcare providers with a rotator cuff problem be initially managed with non-operative treatment and do not need to be referred for an ultrasound, a MRI, or to a surgeon immediately (Figs. 2 and 3). An exception to this guideline should be made for patients presenting with large acute tears of 2 or more rotator cuff tendons (>3 cm) that have been confirmed with diagnostic imaging (preferably confirmed with ultrasound). This category of patient requires surgical intervention and should be seen by a surgeon within 4 weeks of seeking care (Fig. 2). Many studies have demonstrated success in treating patients conservatively with a non-operative program [55–59]. This guideline highlights the importance of early non-operative treatment for these patients [56]. Surgery is an invasive procedure, associated with additional risks, and does not always result in a successful outcome of rotator cuff pathology [60–62]. Surgery is not always the best option for patients, especially patients that have an aversion to the idea of surgical intervention. A significant proportion of patients referred to a surgeon often do not know that non-operative treatment can be an alternative to surgery. In these cases, patients should first be treated with a non-operative program and not referred to a surgeon. Prescription and adherence to an early rehabilitation program has the potential to result in successful treatment of rotator cuff pathology and can reduce utilization of healthcare resources, which ultimately saves costs to both the patient and healthcare system. This will also help to reduce inappropriate surgical referrals. Patient reported outcome measures, such as the Rotator Cuff Quality of Life Index [63] or the Western Ontario Rotator Cuff Index [64], should be used throughout the clinical management algorithm to assess health status and determine the success of treatment.

### Limitations

One criticism of using the modified Delphi method is the loss of subject anonymity in the voting process. Subject anonymity can reduce the effects of dominant individuals, and reduce manipulation or coercion to conform to certain viewpoints [65, 66]. Absence of a face-to-face meeting, however, may deprive experts from exchanging important information, such as clarification of reasons for disagreements [67]. The final meeting allows for an attempt to seek clarification in order to reach consensus, or to generate alternatives for and against best practice [68]. This helps policy makers and stakeholders to make the most appropriate choices. Modified Delphi methodology has since been used by many studies to generate discussion around topics that do not initially meet consensus and is an effective method for addressing clinical problems, which tend to be multi-factorial and complex. Secondly, consensus statements were not supported by level 1 studies; however, it is unlikely that large, well-designed randomized clinical trials relating to diagnosis, physical examination, investigations, or treatment of rotator cuff pathology will be published in the immediate future. In addition, strong recommendations are often provided where there is consistent results from level 2 and 3 studies, and one should not always assume that level 1 studies provide higher quality than level 2 [69]. Thirdly, consensus methods contain certain methodological issues such as bias in the selection of participants or that participants may feel compelled to conform to the group view [70, 71]. For example, there were no radiologists chosen as part of the expert panel who may not agree with the recommended imaging guidelines. Patients were also not chosen as part of the expert panel. Consensus methods, nonetheless, provide a useful way of identifying and measuring uncertainty in medical and health services research, and is increasing in validity for developing clinical guidelines [20–25, 72]. Finally, only 11 experts could attend the face-to-face meeting, in which one expert was only able to participate for half of the meeting and had to leave due to conflicting obligations. Therefore, the results reached in the third round may be biased in favour of the experts that attended the meeting. This bias was minimized, however, because the results from the face-to-face meeting were summarized and distributed to the entire group for final remarks. The final document, including the clinical management algorithms, reached unanimous agreement by the group.

## Conclusion

The purpose of this study was to provide guidance on the diagnosis and treatment of patients with rotator cuff pathology for all clinicians who are likely to diagnose and manage patients with rotator cuff pathology. The purpose is to standardize care and assist in clinical decision-making for any healthcare professional in a primary, secondary, and tertiary healthcare setting, and should not be interpreted as the only course of patient management. The guideline is also meant to accomplish the following: 1) improve the accuracy and efficiency of diagnosing rotator cuff tears, 2), reduce inappropriate use of ancillary tests such as magnetic resonance imaging, and 3) increase the early adoption of appropriate therapeutic rehabilitative exercises. This guideline serves as the first step to informing policy-makers about ideal standards of quality care. The next step is to compare the current state of care for patients presenting to the healthcare system with rotator cuff pathology to the ideal state, represented by this guideline. This will identify gaps in diagnosis and treatment with the ultimate goal of proposing a solution that can help narrow the gap between the ideal state and the current state. This guideline will be periodically reviewed to ensure consensus remains consistent with current medical literature and national guidelines.

## Additional file

Additional file 1: Description of the Clinical Pathway Algorithms for Patients Presenting with Rotator Cuff Pathology describes in detail the decision tree approaches to healthcare treatment for patients presenting with acute, chronic, and acute-on-chronic rotator cuff tears. (PDF 65 kb)
