# Supplementary material for: Using the modified Delphi method to establish clinical consensus for the diagnosis and treatment of patients with rotator cuff pathology
Source: BMC Med Res Methodol. 2016 May 20;16:56. doi: 10.1186/s12874-016-0165-8 (PMC4875724; doi:10.1186/s12874-016-0165-8)
Supplement: Additional file 1: — Description of the Clinical Pathway Algorithms for Patients Presenting with Rotator Cuff Pathology describes in detail the decision tree approaches to healthcare treatment for patients presenting with acute, chronic, and acute-on-chronic rotator cuff tears. (PDF 65 kb) [file 12874_2016_165_MOESM1_ESM.pdf]

ADDITONAL FILE 1: Description of the Clinical Pathway Algorithms for Patients Presenting with Rotator Cuff Pathology

Clinical Algorithm for Patients Presenting with an Acute Rotator Cuff Tear

The clinical pathway algorithm for a patient presenting with an acute rotator cuff tear is presented in Figure 2. An acute rotator cuff tear was defined by the expert panel as a patient who presents with a discrete traumatic episode resulting in an injury to a previously asymptomatic shoulder. The panel recommended that patients be managed using the acute clinical care pathway if they meet the following inclusion criteria: active and high-functioning; asymptomatic prior to the event (no previous history of shoulder problems); a discrete traumatic tear attributable to a specific event or mechanism of injury; experiencing loss of function including an inability to lift the arm; and less than 65 years of age (Figure 2). The first step in the acute clinical care pathway begins when a patient decides to seek care for their injury and enters the primary healthcare system. At the time of their first appointment (T_1), the primary care practitioner should obtain a history using the screening and diagnostic questions and perform a physical examination as per the consensus statements presented in Table 1. The panel then recommended that a standard shoulder x-ray series (*i.e.*, true anteroposterior view, axillary view, and trans-scapular lateral view) be ordered at the initial primary care consultation to rule out fractures and aid in the diagnoses, treatment, and management of the presenting problem. If the x-rays reveal a fracture, the patient should be referred to an orthopaedic surgeon within one week from the injury (ideally same day) and receive surgery if deemed necessary by the treating surgeon. If the primary care practitioner has been appropriately trained to assess and manage a patient who presents following a traumatic shoulder injury, and the x-rays are negative for

fracture, then the primary care practitioner can continue to manage the patient. If the primary care practitioner does not feel confident in their assessment skills, then they should refer the patient to an expert (*e.g.,* sport medicine physician or non-physician expert) who has been appropriately trained to assess the shoulder. The first appointment with an expert (T_2) should take place within two weeks of the referral. An ultrasound can be ordered either by the primary care physician or the expert to confirm rotator cuff pathology. If the ultrasound confirms that only a single rotator cuff tendon is involved, then non-operative treatment can be prescribed for the patient. Non-operative treatment should consist of a 12 week physical therapy program (home or supervised). If the patient is not able to achieve a pain-free status with improved range-of-motion after six weeks of physical therapy, they should receive additional means of pain control (*i.e.,* the use of oral non-steroidal anti-inflammatory medications and/or injectable corticosteroids into the glenohumeral joint or subacromial bursa depending on the site of tendon tear), and continue with physical therapy for another six weeks. If non-operative treatment fails to improve patient outcomes after 12 weeks, the patient can be referred to an orthopaedic surgeon. The first appointment with a surgeon (T_3) should occur within four weeks of the referral. If the surgeon and patient both decide that surgery is the best option, the patient should receive surgery within six weeks of making the decision. If the ultrasound confirms that two or more rotator cuff tendons are involved (*i.e.,* 3-5cm tear), then the patient should be referred to a surgeon (to be seen within four weeks of the referral and proceed to surgery within six weeks of the surgical consult). All patients undergoing surgery should be prescribed a post-operative rehabilitative program.

Clinical Algorithm for Patients Presenting with a Chronic Rotator Cuff Tear

The clinical pathway algorithm for a patient presenting with chronic rotator cuff pathology is presented in Figure 3. A chronic rotator cuff tear was defined as a patient that presents with shoulder pain of insidious or gradual onset or resulted from a previous traumatic episode. These patients should be managed using the chronic clinical care pathway if they present with pain, weakness, and/or altered function (Figure 3). The first step in the chronic clinical care pathway also begins when a patient decides to seek care for their shoulder problem and enters the primary health system. At the time of their first appointment (T_1), the primary care practitioner should take a history using the screening and diagnostic questions and perform a physical examination as per the consensus statements presented in Table 1. Fractures are not typically associated with chronic rotator cuff pathology; however, a standard x-ray series of the shoulder should still be obtained at the initial primary care consultation to help assess degenerative changes that may be present. If the primary care practitioner has been appropriately trained to assess and manage such shoulder pathology, then the primary care practitioner can begin to treat the patient using the non-operative treatment protocol described in the acute pathway. If the primary care practitioner does not feel confident in their assessment skills, then they should refer the patient to an expert (*e.g.,* sport medicine physician or non-physician expert) who has been appropriately trained to assess and manage shoulder problems. The first appointment with an expert (T_2) should take place within two weeks of the referral. An ultrasound need only be ordered, either by the primary care physician or the expert, if the patient fails non-operative management. If non-operative treatment fails to improve patient outcomes after 12 weeks, and the ultrasound confirms presence of rotator cuff pathology, the patient can be referred to a surgeon [34]. The first appointment with a surgeon (T_3) should occur within six to 12 weeks after completion of the non-operative program. If the surgeon and patient both decide that surgery is the best option, the

patient should receive surgery within 12 weeks of making the decision. If the ultrasound does not confirm the presence of rotator cuff pathology, but the presence of another underlying pathology, the pathology should be treated according to current clinical research and practice. All patients undergoing surgery should be prescribed a post-operative rehabilitative program.

Clinical Algorithm for Patients Presenting with an Acute-on-Chronic Rotator Cuff Tear

An acute-on-chronic rotator cuff problem was defined by the panel as a patient with pre-existing rotator cuff pathology who experiences a traumatic episode to the ipsilateral shoulder. It was agreed that these patients should also be managed using the chronic clinical care pathway if they presented with pain, weakness, and/or altered function (Figure 3).
